# Orbital apex syndrome in noninvasive fungal rhinosinusitis: a case report

**DOI:** 10.1093/jscr/rjaf766

**Published:** 2025-09-28

**Authors:** Zhongju Yin, Xiaoyan Hu, Shuang Liu, Liang Jiang

**Affiliations:** Department of Otolaryngology Head and Neck Surgery, Affiliated Hospital of Southwest Medical University, No. 25 Taiping Street, Jiangyang District, Luzhou City, Sichuan Province 646000, PR China; Department of Pathogen Biology, School of Basic Medicine, Southwest Medical University, No. 25 Taiping Street, Jiangyang District, Luzhou City, Sichuan Province 646000, PR China; Public Center of Experimental Technology of Pathogen Biology Technology Platform, Southwest Medical University, No. 25 Taiping Street, Jiangyang District, Luzhou City, Sichuan Province 646000, PR of China; Department of Otolaryngology Head and Neck Surgery, Deyang People's Hospital, No. 173, Section 1, Taishan North Road, Jingyang District, Deyang City, Sichuan Province 618000, PR of China; Department of Otolaryngology Head and Neck Surgery, Affiliated Hospital of Southwest Medical University, No. 25 Taiping Street, Jiangyang District, Luzhou City, Sichuan Province 646000, PR China

**Keywords:** orbital apex syndrome (OAS), noninvasive fungal rhinosinusitis (NIFRS), fungal rhinosinusitis, functional endoscopic sinus surgery (FESS)

## Abstract

Noninvasive fungal rhinosinusitis is the most common type of fungal sinusitis, but it rarely leads to orbital apex syndrome. In this case study, we present a 44-year-old female who developed headache and sudden loss of vision in the left eye. Examination revealed ptosis, complete visual loss, and total ophthalmoplegia in the left eye. Imaging showed a fully opacified mass in the left orbital apex and spheno-ethmoidal recess, involving the adjacent ophthalmic artery, optic nerve, and extraocular muscles. The patient was diagnosed with orbital apex syndrome secondary to noninvasive fungal rhinosinusitis. She received a 3-day course of methylprednisolone without improvement, followed by functional endoscopic sinus surgery. Histopathology confirmed a fungal ball (aspergilloma) without tissue invasion. The patient was hospitalized for 2 weeks, treated with perioperative antibiotics, but did not receive systemic antifungal therapy. At 3 months follow-up, headache, proptosis, and pain had resolved, but vision loss and ptosis did not improve significantly. Early recognition and timely surgical intervention are crucial in preventing irreversible complications of noninvasive fungal rhinosinusitis.

## Introduction

Fungal rhinosinusitis (FRS) is a relatively common infection in rhinology. Due to the widespread use of antibiotics, glucocorticoids, and immunosuppressants, its incidence has increased in recent years. FRS is classified into invasive fungal rhinosinusitis (IFRS) and noninvasive fungal rhinosinusitis (NIFRS). IFRS, due to its mucosal and bony invasion, may spread through blood vessels or nerves to adjacent structures, often causing serious complications within a short time. In contrast, NIFRS is usually confined to the sinus cavity. However, with disease progression, it may still lead to complications such as facial swelling, periorbital edema, pain, and in some cases, orbital or optic nerve involvement resulting in vision loss or blindness. These complications can be as serious as those of IFRS. Here, we report a case of NIFRS presenting with orbital apex syndrome (OAS) in a 44-year-old female.

## Case presentation

A 44-year-old Chinese female presented to the Ophthalmology Department with a 1-month history of headache and left eye pain, followed by a one-week history of sudden decreased vision and ophthalmoplegia in the left eye. She had poorly controlled diabetes mellitus and hypertension. Clinical examination showed left eyelid ptosis, complete visual loss, and total ophthalmoplegia in the left eye, while the right eye was normal. Glycosylated hemoglobin was elevated at 14%.

**Figure 1:**
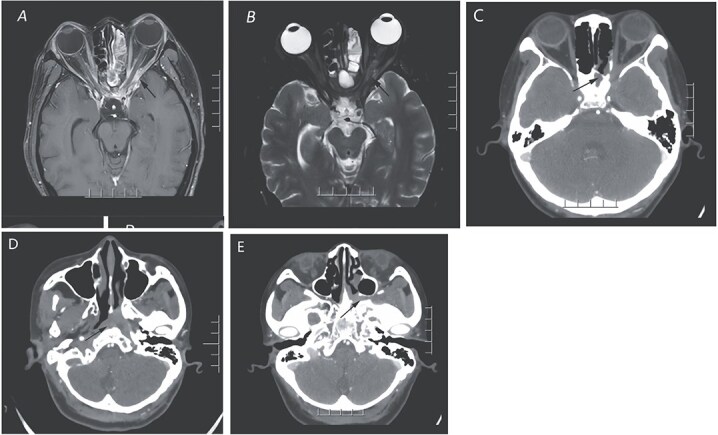
The orbital enhanced MRI scan (A and B) illustrates the presence of an enhancing inflammatory phlegmon that extends from the left sphenoid sinus through the orbital apex and involves the extraocular muscles of the left orbit. Additionally, there is an abnormal swelling of the left optic nerve. In panel C, the CT scan of the paranasal sinuses in the coronal view reveals a mass in the sphenoid sinus and posterior ethmoid sinus, along with bone destruction and a suspicious soft tissue mass affecting the left orbital apex. Panels D and E display the extension of the mass into the left pterygopalatine fossa and the presence of a suspicious soft tissue mass in the left nasopharynx, indicated by arrows.

Magnetic resonance imaging (MRI) revealed a mass in the left orbital apex and spheno-ethmoidal recess, involving the adjacent optic nerve, ophthalmic artery, and extraocular muscles (Fig. 1A and B). CT scan demonstrated opacification of the sphenoid and posterior ethmoid sinuses with bony erosion, a mass extending into the orbital apex, pterygopalatine fossa, and nasopharynx (Fig. 1C–E). These findings were suggestive of an inflammatory lesion, but neoplastic disease could not be excluded.

**Figure 2:**
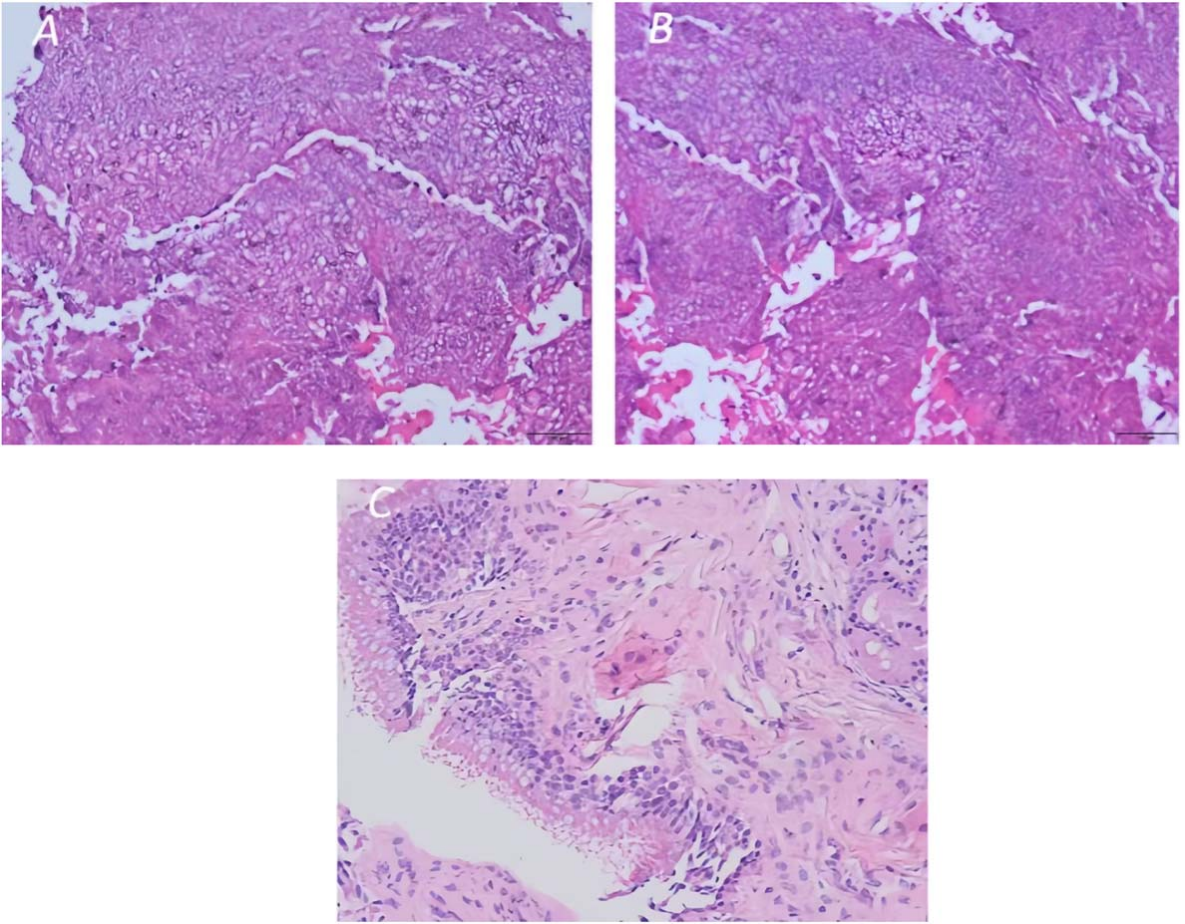
(A and B) Histopathology image of a fungal ball (aspergilloma) characterized by radiating septate hyphae and (C) chronic inflammation of the sinus mucosa (original magnification ×200).

The patient received intravenous methylprednisolone for 3 days, but her pain and ptosis worsened. Following consultation with the otorhinolaryngologists, the left sphenoid sinus, orbital apex, nasopharynx, and pterygopalatine fossa mass were considered. Although the nature was unknown, there was some evidence of bone destruction on CT scan. Tumor could not be ruled out, so it was recommended to transfer her to the Otorhinolaryngology Department for further treatment. On 27 July 2023 (Day 10 of hospital stay), she underwent functional endoscopic sinus surgery (FESS). Intraoperatively, yellow-brown fungal balls were found completely occupying the sphenoid sinus. Frozen sections confirmed fungal elements. Histopathology of the surgical specimen revealed a fungal ball (aspergilloma) with septate hyphae (Fig. 2A and B), while the mucosa showed inflammation but no tissue invasion (Fig. 2C).

The patient remained hospitalized for 2 weeks and was treated with intravenous cefuroxime perioperatively. She did not receive systemic antifungal therapy. At discharge, her headache and orbital pain had improved. At the 3-month follow-up, headache, pain, and proptosis had resolved, but there was no significant recovery of vision or ptosis.

## Discussion

OAS due to FRS is uncommon, and its occurrence in NIFRS is exceedingly rare. The syndrome can involve the optic nerve, cranial nerves III, IV, VI, and the ophthalmic division of V. In this case, progressive unilateral vision loss unresponsive to medical therapy required urgent surgical decompression.

FESS revealed a fungal ball in the sphenoid sinus, consistent with NIFRS. Histopathology confirmed no tissue invasion. While IFRS often causes rapid orbital complications due to aggressive invasion, NIFRS is typically confined to the sinus cavity [1]. However, in this patient, a defect in the medial wall of the optic canal was visible, suggesting indirect extension and inflammation of adjacent structures. This likely contributed to optic nerve involvement.

Literature indicates that vision loss in NIFRS can result from the close proximity of the sphenoid sinus to the optic nerve, direct compression, or central retinal artery occlusion [2–4]. In this patient, no significant compression was seen, and the mechanism may have been inflammation and edema transmitted through congenital or acquired dehiscence of the optic canal wall.

Preoperative imaging in this case was atypical, lacking the characteristic calcified densities often seen in fungal sinusitis [5]. CT and MRI primarily suggested chronic inflammation, contributing to diagnostic delay. Visual outcome was poor, consistent with reports that surgery offers limited benefit in cases of complete vision loss prior to intervention.

## Conclusion

NIFRS presenting with OAS is rare and easily misdiagnosed as an isolated ophthalmic disorder. Lack of typical imaging findings and insufficient awareness among clinicians may delay diagnosis and treatment. Multidisciplinary evaluation and early imaging are crucial for timely recognition. Prompt surgical intervention, such as FESS, is essential to prevent irreversible complications, although visual recovery may be limited if optic nerve damage is advanced.

## Data Availability

Not applicable.
